# Cellular androgen content influences enzalutamide agonism of F877L mutant androgen receptor

**DOI:** 10.18632/oncotarget.9816

**Published:** 2016-06-03

**Authors:** Daniel J. Coleman, Kathryn Van Hook, Carly J. King, Jacob Schwartzman, Robert Lisac, Joshua Urrutia, Archana Sehrawat, Josha Woodward, Nicholas J. Wang, Roman Gulati, George V. Thomas, Tomasz M. Beer, Martin Gleave, James E. Korkola, Lina Gao, Laura M. Heiser, Joshi J. Alumkal

**Affiliations:** ^1^ OHSU Knight Cancer Institute, Oregon Health & Science University, Portland, Oregon, U.S.A; ^2^ Department of Biomedical Engineering, Oregon Health & Science University, Portland, Oregon, U.S.A; ^3^ Division of Public Health Sciences, Fred Hutchinson Cancer Research Center, Seattle, Washington, U.S.A; ^4^ The Vancouver Prostate Centre and Department of Urologic Sciences, University of British Columbia, Vancouver, British Columbia, Canada

**Keywords:** genitourinary cancers: prostate, hormone signaling and inhibitors, regulation of gene expression in drug resistance, androgen receptor mutations, BET bromodomain inhibition

## Abstract

Prostate cancer is the most commonly diagnosed and second-most lethal cancer among men in the United States. The vast majority of prostate cancer deaths are due to castration-resistant prostate cancer (CRPC) – the lethal form of the disease that has progressed despite therapies that interfere with activation of androgen receptor (AR) signaling. One emergent resistance mechanism to medical castration is synthesis of intratumoral androgens that activate the AR. This insight led to the development of the AR antagonist enzalutamide. However, resistance to enzalutamide invariably develops, and disease progression is nearly universal. One mechanism of resistance to enzalutamide is an F877L mutation in the AR ligand-binding domain that can convert enzalutamide to an agonist of AR activity. However, mechanisms that contribute to the agonist switch had not been fully clarified, and there were no therapies to block AR F877L. Using cell line models of castration-resistant prostate cancer (CRPC), we determined that cellular androgen content influences enzalutamide agonism of mutant F877L AR. Further, enzalutamide treatment of AR F877L-expressing cell lines recapitulated the effects of androgen activation of F877L AR or wild-type AR. Because the BET bromodomain inhibitor JQ-1 was previously shown to block androgen activation of wild-type AR, we tested JQ-1 in AR F877L-expressing CRPC models. We determined that JQ-1 suppressed androgen or enzalutamide activation of mutant F877L AR and suppressed growth of mutant F877L AR CRPC tumors *in vivo*, demonstrating a new strategy to treat tumors harboring this mutation.

## INTRODUCTION

Prostate cancer is the most commonly diagnosed and second-most lethal cancer in men in the United States [1]. The vast majority of prostate cancer deaths are due to castration-resistant prostate cancer (CRPC) – the lethal form of the disease that has progressed despite therapies that interfere with activation of the androgen receptor (AR) [1]. Recent work demonstrates that CRPC cells are capable of synthesizing their own androgens and that these intratumoral androgen levels are sufficient to maintain AR function [2, 3]. These discoveries led to the development of the second generation anti-androgen enzalutamide that suppresses CRPC tumor growth in pre-clinical models [4]. Recently, two phase III clinical trials demonstrated a significant overall survival benefit with enzalutamide treatment vs. placebo, leading to enzalutamide's approval for CRPC patients [5, 6]. However, many patients do not respond to enzalutamide treatment, and disease progression is nearly universal. Thus, understanding mechanisms that contribute to enzalutamide resistance is crucial for the development of more effective treatment strategies.

Previously, several groups identified mutations in the AR ligand binding domain (LBD) that convert first-generation anti-androgens such as bicalutamide and flutamide to AR agonists rather than antagonists [7, 8]. Clinically, discontinuation of these first-generation anti-androgens leads to so-called “anti-androgen withdrawal” effects (PSA declines) in as many as 25% of patients [9]. Several groups have detected F877L mutations (alternatively described as F876L based on older genomic builds) in the AR LBD following chronic treatment of prostate cancer cell lines with enzalutamide or other second-generation anti-androgens such as ARN-509 [10–12]. AR F877L mutations confer resistance to enzalutamide treatment and have been shown to cause an antagonist to agonist switch in some cases [10–12]. Approximately 5-10% of patients harbor F877L mutations after treatment with novel anti-androgens such as enzalutamide or ARN-509, demonstrating the clinical relevance of this mutation [11, 13]. However, anti-androgen withdrawal effects after discontinuing enzalutamide are only observed in a small percentage of patients [14]. Mechanisms that contribute to enzalutamide agonism of AR function are not fully understood, and it is not clear why enzalutamide anti-androgen withdrawal effects are rarely observed in the clinic. Further, there are currently no effective treatments to suppress mutant F877L AR function.

In this study, we used cellular models to identify molecular mechanisms that contribute to enzalutamide agonism of mutant F877L AR. We determined that enzalutamide treatment of AR F877L-expressing cells led to greater agonistic effects when the cells were cultured in conditions with low androgen ligands. This suggests that the androgen content of the cell may determine whether enzalutamide has the capacity to activate mutant F877L AR. Further, we found that enzalutamide activation of mutant F877L AR leads to induction of a similar, albeit smaller, set of genes that are activated by the natural AR ligand dihydrotestosterone (DHT). This suggests that DHT is a more potent activator of AR function.

Finally, prior work showed that inhibition of the BET bromodomain family of chromatin readers is an effective strategy to suppress androgen ligand activation of wild-type AR [15]. We determined that BET bromodomain inhibition also interferes with either androgen or enzalutamide activation of mutant F877L AR and suppresses CRPC cell viability *in vitro* and *in vivo.* Thus, BET bromodomain inhibition is a promising strategy to block AR F877L function irrespective of whether the AR ligand is androgens or enzalutamide.

## RESULTS

### Androgens influence enzalutamide agonism of mutant F877L AR

**Figure 1:**
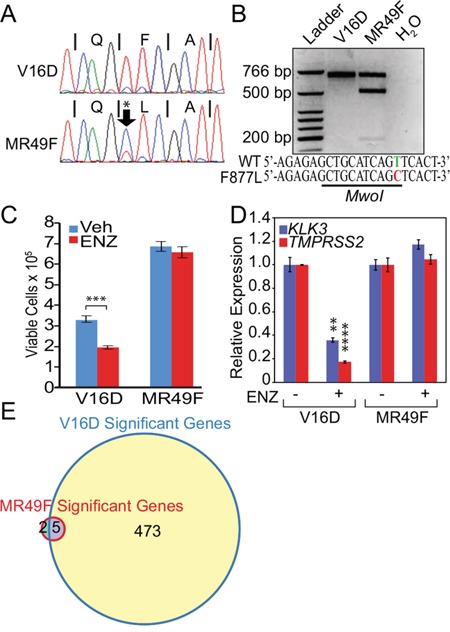
Mutant F877L AR-expressing MR49F cells are resistant to enzalutamide, but agonist effects are not seen in androgen-replete conditions **A.** Sanger sequencing trace of a 624 bp PCR product amplified from parental V16D or MR49F cell line genomic DNA, containing the region encoding the AR LBD. A T→C mutation corresponding to the F877L mutation was detected specifically in MR49F cells. **B.** Restriction digests of the PCR products from V16D or MR49F cells with MwoI. This enzyme only digests this DNA fragment if it harbors a T→C F877L mutation. Retention of an upper, 624 bp band in the MR49F digest demonstrates that this mutation is heterozygous. **C.** Trypan Blue assay of cell viability. V16D and MR49F cells were grown in full serum and were treated with vehicle or 10 μM enzalutamide for five days. Data are means of three biological replicates; error bars represent standard deviations. *** = p≤0.001, unpaired 2-tailed t-test. **D.** RT-qPCR of canonical AR target genes *KLK3* and *TMPRSS2* following 24 hour treatment with vehicle or 10 μM enzalutamide. Data are mean RQ (ΔΔCt method) of three biological replicates; positive and negative error bars represent standard error of the mean (SEM). ** = p≤0.01, **** = p≤0.0001, unpaired 2-tailed t-test with Benjamini-Hochberg False Discovery Rate. Comparisons were made between vehicle and enzalutamide treated samples. **E.** Venn diagram of RNA-seq transcriptional changes after 24 hour enzalutamide treatment (10 μM) demonstrating 478 significant differentially-expressed genes in parental V16D cells but only seven in resistant MR49F cells. Expression data per gene represent the mean, log2-transformed FPKM values of three biological replicates. After filtering based on variance, we used a t-test to determine significant differentially-expressed genes in the enzalutamide vs. vehicle-treated conditions (FDR-adjusted p-value ≤ 0.05).

In order to study the problem of acquired enzalutamide resistance, several groups have chronically treated prostate cancer cell lines *in vitro* or *in vivo* with enzalutamide. One example is the MR49F cell line that was derived after LNCaP cells were implanted in castrated mice and treated chronically with enzalutamide [16, 17]. MR49F cells have been previously found to contain an AR F877L mutation [18] and a nearly full copy number gain of the AR versus their parental LNCaP CRPC derivative cell line called V16D [19]. To confirm the mutational status of these cell lines, we used PCR to amplify a 624 bp region encoding the AR LBD in both MR49F and V16D cells and then performed Sanger sequencing on the products. Sequencing confirmed a T→C mutation corresponding to the mutant F877L AR in MR49F cells but not in the parental V16D line (Figure 1A). To determine if background observed in the V16D sequencing trace (Figure 1A) was due to this mutation being present at low frequency in the parental V16D cells, we performed a restriction digest on the PCR products. The T→C base pair change that corresponds to the F877L mutation results in the generation of an MwoI restriction site. No digestion with MwoI was observed in the PCR products amplified from V16D cells (Figure 1B). On the other hand, MwoI digestion of the PCR product amplified from MR49F cells led to digestion products of 489 and 187 base pairs (bp). The presence of the upper, undigested 624 bp band in the MR49F PCR product indicates that the F877L mutation in MR49F cells is heterozygous (Figure 1B). Overall, these results demonstrate that the AR F877L mutation is not readily identifiable in parental V16D cells and suggests that this mutation may be acquired with enzalutamide resistance, which matches prior reports [10, 11].

Next, we cultured V16D cells or MR49F cells in growth media supplemented with fetal bovine serum (FBS). Importantly, prostate cancer cells are capable of metabolizing the testosterone found in FBS into dihydrotestosterone (DHT) at concentrations similar to those found in CRPC tumors (~1–10 nM) that promote AR function and CRPC growth [20, 21]. We treated both of these cell lines with enzalutamide (10 μM) and measured cell viability. Enzalutamide treatment reduced viability of V16D cells but did not change viability of MR49F cells (Figure 1C). Importantly, while the MR49F cells were resistant to treatment, we observed no agonistic effect on cell growth despite the fact that MR49F cells harbor an AR F877L mutation (Figure 1C). We then measured the effect of enzalutamide treatment on canonical AR target genes in V16D and MR49F cells. Enzalutamide treatment suppressed expression of *KLK3* and *TMPRSS2* in V16D cells (Figure 1D). However, enzalutamide treatment of MR49F cells did not lead to significant changes in expression of these genes. Importantly, no significant agonistic effects on gene expression were seen (Figure 1D).

To more globally examine the effect of enzalutamide on gene expression, we performed RNA-seq on MR49F or V16D cells treated with vehicle or enzalutamide. Enzalutamide treatment of V16D cells led to 478 differentially-expressed genes (Figure 1E, Supplementary Figure S1A, also see Supplementary Gene Lists corresponding to these figures); gene set enrichment analysis (GSEA) showed significant enrichment for a signature of canonical AR target genes described previously [15] (Supplementary Figure S2A, also see Supplementary Gene List corresponding to this figure). On the other hand, enzalutamide treatment of MR49F cells only resulted in seven differentially-expressed genes (Figure 1E, Supplementary Figure S1B, also see Supplementary Gene Lists corresponding to these figures). In examining the RNA-seq data for AR transcripts, we determined that 61% of the MR49F RNA-seq reads mapping to the AR LBD contained the cytosine nucleotide corresponding to the F877L mutation while the remainder corresponded to the wild-type allele. Conversely, the wild-type allele was observed in 100% of reads in V16D cells. This confirms results in Figure 1 that the F877L mutation is heterozygous in MR49F cells and that this mutation is either not present or is very rare in the parental V16D line. Overall, the above results demonstrate that enzalutamide does not confer a growth advantage or activate AR transcriptional activity when AR F877L-expressing cells are cultured with androgen levels similar to CRPC tumors.

To confirm that MR49F cells were still AR-dependent, we grew these cells in FBS supplemented with enzalutamide and then suppressed AR with RNAi. AR knockdown significantly decreased cell viability and expression of AR target genes (Supplementary Figure S3). These data demonstrate a continued dependence of AR in enzalutamide-resistant MR49F cells despite enzalutamide treatment.

**Figure 2:**
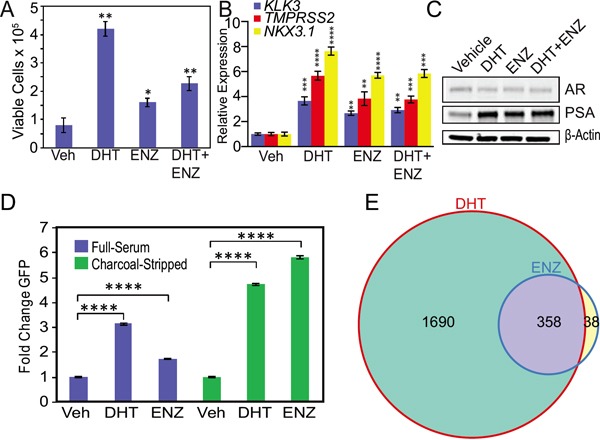
Androgen depletion accentuates enzalutamide agonism of mutant F877L AR **A.** Trypan blue cell viability assay of enzalutamide-resistant MR49F cells cultured with charcoal-stripped serum. Cells were grown for four days in medium containing charcoal-stripped serum, and then cells were treated with fresh medium containing either vehicle, 10 μM enzalutamide, 1 nM DHT, or the combination for six days. Treatment media was changed on day three. Data are means of three biological replicates; error bars represent standard deviations. * = p≤0.05, ** = p≤0.01, unpaired 2-tailed t-test. Comparisons are to vehicle treatment. **B.** RT-qPCR was used to quantify mRNA expression of canonical AR targets *KLK3*, *TMPRSS2*, and *NKX3.1* in MR49F cells cultured with charcoal-stripped serum. Cells were grown for three days in medium containing charcoal-stripped serum, and then cells were treated with fresh medium containing either vehicle, 10 μM enzalutamide, 1 nM DHT, or the combination for 24 hours. Data are mean RQ (ΔΔCt method) of three biological replicates; positive and negative error bars represent standard error of the mean (SEM). ** = p≤0.01, *** = p≤0.001, **** = p≤0.0001, unpaired 2-tailed t-test. Comparisons are to vehicle treatment. **C.** Western blots of protein lysates from experiments above in (B) were used to measure PSA protein expression in MR49F cells. **D.** LNCaP cells stably overexpressing ectopic F877L AR and a probasin promoter GFP reporter (LNCaP Pb. EGFP ^ARF877L^) were grown in full serum or charcoal-stripped serum and then treated with fresh medium containing either vehicle, 1 nM DHT, or 10 μM enzalutamide for six days. Treatment media was changed on day three. For the charcoal-stripped condition, cells were switched to medium with charcoal-stripped serum for 24 hours prior to drug treatment. GFP expression was measured with flow cytometry. Data are means of three biological replicates; error bars represent SEM. **** = p≤.0001, unpaired 2-tailed t-test. **E.** MR49F cells were grown in medium containing charcoal-stripped serum for three days, and then treated with fresh medium containing either vehicle, 1 nM DHT or 10 μM enzalutamide for 24 hours prior to harvest for RNA-seq. Venn diagram of RNA-seq data demonstrating overlap of genes induced by DHT or enzalutamide treatment. Expression data per gene represents the mean read count values of three biological replicates. After variance stabilizing the data using DESeq, we used a t-test to determine significant differentially-expressed genes in the drug-treated vs. vehicle-treated conditions (FDR-adjusted p-value ≤0.05).

Because of these results, we hypothesized that androgens may compete for enzalutamide activation of mutant F877L AR. Therefore, we cultured MR49F cells in androgen-depleted conditions using medium supplemented with charcoal-stripped FBS, which contains ~80% less total testosterone than medium supplemented with standard FBS [20]. We then added either vehicle, 1 nM DHT (based on prior reports that this level of DHT is commonly found in CRPC metastases) [21], 10 μM enzalutamide, or both in combination. DHT increased cell growth as expected (Figure 2A). Enzalutamide alone also increased cell growth under these androgen-depleted conditions (Figure 2A). The combination of DHT and enzalutamide also increased cell growth, though this effect was attenuated compared to DHT alone (Figure 2A). Further, DHT alone, enzalutamide alone, or the combination in androgen-depleted conditions also increased expression of canonical AR targets: *KLK3*, *TMPRSS2*, and *NKX3.1* (Figure 2B) and increased protein levels of PSA encoded by the *KLK3* gene (Figure 2C). This suggests that the cellular androgen content influences enzalutamide's ability to activate mutant F877L AR.

To determine if the effect of androgen content on enzalutamide agonism of F877L was generalizable, we used LNCaP cells stably overexpressing F877L AR and also an AR-driven probasin promoter GFP reporter construct (LNCaP Pb. EGFP ^ARF877L^, also known as LNCaP-F877L) [12]. We treated cells with enzalutamide or DHT and then performed flow cytometry to quantify GFP expression. Enzalutamide treatment of LNCaP-F877L cells cultured in androgen-replete serum led to a modest induction of AR function as measured by GFP expression (Figure 2D, Supplementary Figure S4A). On the other hand, enzalutamide treatment of LNCaP-F877L cells grown in androgen-depleted, charcoal-stripped serum led to a much greater induction of GFP expression (Figure 2D, Supplementary Figure S4A). This matches the results in MR49F cells. As expected, enzalutamide did not activate GFP expression in LNCaP cells with stable overexpression of wild-type AR and the Pb. EGFP reporter (Supplementary Figure S4B).

### Gene expression changes induced by enzalutamide overlap with those induced by DHT

To more globally examine the effects of DHT or enzalutamide on F877L AR activation, we grew MR49F cells in charcoal-stripped serum, treated the cells with vehicle, 10 μM enzalutamide, or 1 nM DHT, and then performed RNA-seq. Under androgen-depleted conditions, enzalutamide treatment led to 396 significantly differentially expressed genes compared to vehicle treatment. DHT treatment, on the other hand, induced expression changes in 2048 genes, again suggesting that DHT is a more potent agonist than enzalutamide in MR49F cells at the concentrations tested. Both the DHT- and enzalutamide-activated gene sets showed significant enrichment for the aforementioned signature of canonical AR target genes (Supplementary Figure S2B, S2C, also see Supplementary Gene List corresponding to this figure) [15]. This demonstrates that enzalutamide or DHT activation of mutant F877L AR upregulates a transcriptional program similar to wild-type AR.

We next sought to determine whether there was significant overlap between the differentially expressed genes after either DHT or enzalutamide treatment in MR49F cells. Of the 396 genes that were differentially expressed with enzalutamide treatment, 358 were also differentially-expressed with DHT treatment (Figure 2E, also see Supplementary Gene Lists corresponding to this figure), demonstrating that enzalutamide activates many of the same AR target genes as natural AR ligands in F877L AR-expressing cells. Furthermore, because of the much greater number of gene expression changes seen with enzalutamide in androgen-depleted conditions (Figure 2E) vs. androgen-replete conditions (Figure 1E), these results further suggest that the androgen content of the cell is a key determinant of enzalutamide's capacity to act as an agonist of mutant F877L AR.

### BET bromodomain inhibition prevents activation of mutant F877L AR and suppresses CRPC cell viability

There are currently no effective treatments to disrupt mutant F877L AR function. We determined that enzalutamide agonism of mutant F877L AR recapitulates the effects of androgen activation of F877L AR (Figure 2E) and that androgen activation of mutant F877L AR recapitulates activation of wild-type AR (Supplementary Figure S2B, S2C). Therefore, we focused on therapeutic strategies that were previously shown to block androgen activation of wild-type AR transcriptional activation.

**Figure 3:**
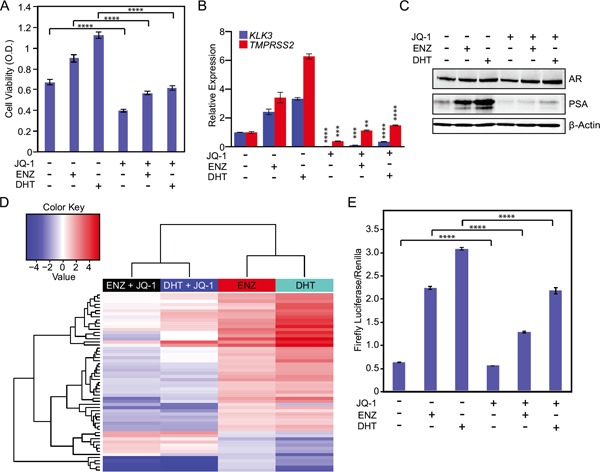
BET bromodomain inhibition prevents activation of mutant F877L AR and suppresses CRPC cell viability in androgen-depleted conditions **A.** MTS cell viability assay of MR49F cells. Cells were grown for three days in charcoal-stripped serum, and then we treated cells with enzalutamide (10 μM) and DHT (1 nM) or vehicle +/− 500 nM JQ-1 for three days. Data are the means of six biological replicates; error bars represent standard deviations. **** = p≤.0001, unpaired 2-tailed t-test. **B.** RT-qPCR to measure mRNA expression of AR target genes in MR49F cells from experiments described in (A). Data are mean RQ (ΔΔCt method) of three biological replicates; positive and negative error bars represent SEM. ** = p≤0.01, *** = p≤0.001, **** = p≤0.0001, unpaired 2-tailed t-test. Comparisons are between respective vehicle and JQ-1 treated groups (ENZ+ vehicle vs. ENZ+JQ-1, etc.). **C.** Western blot was used to measure PSA protein expression in MR49F cells treated as in (A). **D.** Heat map of RNA-seq gene expression changes in MR49F cells treated as in (A). 57 shared enzalutamide and DHT-regulated genes from Figure 2E whose expression is significantly reversed (≥2 fold) by JQ-1 are shown. Expression data per gene represent the mean read count value of three biological replicates. After variance stabilizing the data using DESeq, we used a t-test to determine significant differentially-expressed genes in the JQ-1-treated conditions (average of enzalutamide + JQ-1 and DHT + JQ-1) when compared to vehicle (average of enzalutamide+vehicle and DHT+vehicle). FDR-adjusted p-value ≤0.05. **E.** Luciferase reporter assay of AR function in AR-null PC3 cells transfected with ectopic mutant F877L AR. Cells were cultured in charcoal-stripped serum and transfected with an ectopic AR F877L plasmid, an ARE-4 firefly luciferase reporter plasmid, and a Renilla luciferase control plasmid for 72 hours. Data are means of three biological triplicates; error bars represent standard deviation. **** = p≤0.0001, unpaired 2-tailed t-test.

Prior reports demonstrate that inhibition of BET bromodomain chromatin reader proteins disrupts androgen ligand-induced activation of wild-type AR [15]. Therefore, we determined the effects of BET bromodomain inhibition on mutant F877L AR activation. Co-treatment with the BET bromodomain inhibitor JQ-1 [22] blocked DHT- or enzalutamide-induced growth of MR49F cells cultured in androgen-depleted conditions (Figure 3A) and attenuated expression of downstream AR target genes (Figure 3B) and PSA protein (Figure 3C). Importantly, a much higher concentration of JQ-1 was required to reduce cell viability in MR49F cells grown in androgen-depleted serum vs. androgen-replete serum (Supplementary Figure S5). This is in keeping with a prior report that suggests that suppression of ligand-dependent AR signaling is a critical contributor to the anti-tumor activity of JQ-1 in prostate cancer cells [15].

To more globally examine the effects of BET bromodomain inhibition on AR transcriptional function, we performed RNA-seq on samples from MR49F cells grown in androgen-depleted conditions and stimulated with vehicle, DHT, or enzalutamide with or without JQ-1. As described above, 358 conserved genes were significantly differentially expressed (at any fold change) in response to enzalutamide and DHT treatment (Figure 2E). JQ-1 co-treatment with either DHT or enzalutamide resulted in substantial reversal of the expression changes induced by DHT or enzalutamide alone. To home in on the genes and pathways most strongly changed by JQ-1 co-treatment, we identified the genes that showed at least a 2-fold change in expression with JQ-1 co-treatment as compared to treatment with either enzalutamide or DHT alone. Of the 358 conserved DHT- and enzalutamide-regulated genes, 57 genes met this criteria (Figure 3D, also see Supplementary Gene List corresponding to this figure). There was a significant enrichment of the previously described AR signature in these 57 genes (p ≤ 6.14 × 10^−9^) [15]. Furthermore, many of the same gene ontology (GO) categories were enriched in the 358 conserved genes after DHT or enzalutamide treatment and in the 57 genes changed most by JQ-1 treatment, demonstrating that JQ-1 blocks enzalutamide or DHT activation of AR function in mutant F877L AR-expressing cells (Supplementary Tables S1 & S2).

MR49F cells harbor both non-mutant and mutant F877L AR alleles. To determine if the JQ-1 treatment effect was independent of effects on wild-type AR, we transfected AR-null PC3 cells with ectopic mutant F877L AR and an ARE4 luciferase reporter of AR function, and then treated them with DHT or enzalutamide +/− JQ-1 [23, 24]. JQ-1 partially abrogated the effects of DHT or enzalutamide on AR reporter activation, demonstrating that JQ-1 blocks F877L function even in the absence of wild-type AR (Figure 3E).

**Figure 4:**
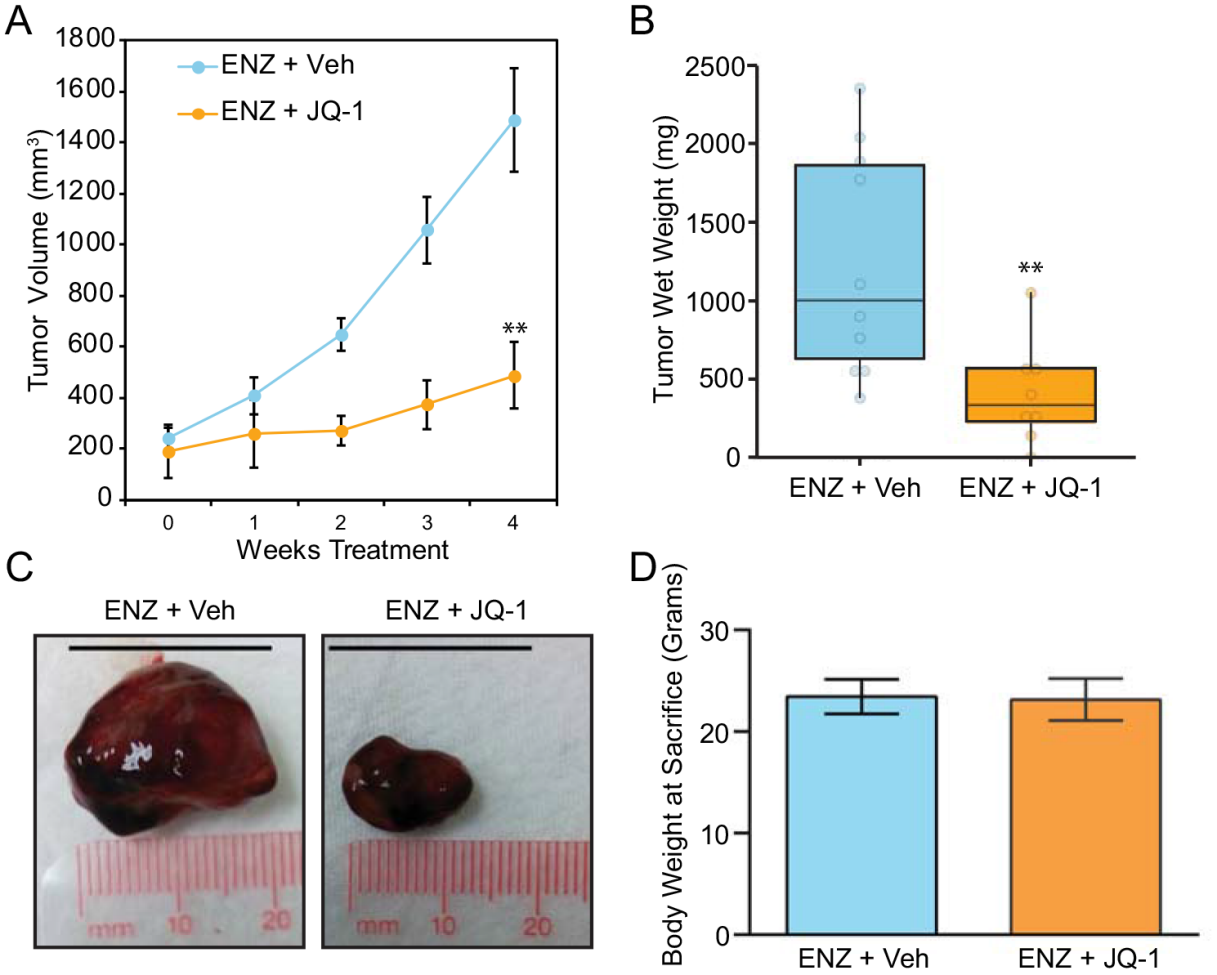
BET bromodomain inhibition suppresses growth of mutant F877L AR xenografts implanted in castrated mice **A.** MR49F cells were implanted in the flanks of castrated, immunocompromised mice. Once tumors reached 100mm^3^, mice were randomly assigned to two groups: Monday-Friday (M-F) treatment with enzalutamide (10 mg/kg) by oral gavage plus vehicle by intraperitoneal injection (n=10 animals) or M-F treatment with enzalutamide (10 mg/kg) + JQ-1 50 mg/kg by intraperitoneal injection (n=eight animals) for four weeks. Tumor volume was measured weekly (length × width caliper readings). Data are mean tumor volumes; error bars represent SEM. 2-sided t-test with equal variance was performed at end point (week 4). **: p ≤ 0.01. **B.** Mice were sacrificed at the conclusion of the study, and tumor wet weights were recorded. 2-sided, 2-sample t-test with Welch's correction was performed. **: p ≤ 0.01. **C.** Representative images of xenografts from each treatment group following sacrifice and removal from mice. Scale bar= 20 mm. **D.** No significant difference in final body weight was observed between the two treatment groups (2-sided t-test with equal variance).

Finally, we sought to determine the tolerability and preliminary anti-tumor efficacy of JQ-1 in an F877L CRPC model *in vivo*. Prior work demonstrated that enzalutamide treatment of MR49F xenografts implanted in castrated mice leads to agonistic growth compared to vehicle treatment [25]. Since this agonist effect was already known, we designed a streamlined, two-armed study to determine if JQ-1 could block enzalutamide-induced growth of MR49F xenografts. We implanted MR49F cells in castrated mice and compared the effect of treatment with enzalutamide alone or enzalutamide + JQ-1. Xenografts grew robustly in mice treated with enzalutamide alone as expected, while enzalutamide + JQ-1 treatment significantly reduced this growth (Figure 4A-4C). There were no differences in animals' weights between the groups (Figure 4D), and no organ toxicity was observed upon visual inspection with the combination, demonstrating the preliminary safety and activity of this combination. Altogether, these results demonstrate that BET bromodomain inhibition interferes with androgen or enzalutamide activation of mutant F877L AR and that BET bromodomain inhibition is a promising therapy to block the growth of enzalutamide-resistant CRPC tumors harboring this mutation.

## DISCUSSION

Despite medical castration, CRPCs are still dependent on the AR [26]. AR amplification is a common feature of CRPC, and upregulation of the androgen synthetic machinery is an emergent resistance mechanism in CRPC tumor cells to overcome androgen deprivation therapies [2, 3]. In recent years, drugs that interfere with these persistent intratumoral androgens have emerged as an effective strategy to combat CRPC. Abiraterone suppresses synthesis of androgens from the adrenal gland and tumor cells via CYP17 inhibition [27–29] and has also been found to interfere directly with ligand binding to the AR [30]. The second-generation anti-androgen enzalutamide competitively inhibits binding of ligands to AR [29, 31]. These drugs improved median survival for CRPC patients in phase III clinical trials [5, 6, 32, 33]. However, resistance to these drugs invariably develops.

One mechanism of resistance to newer AR-targeting agents is the expression of AR splice variants that lack the LBD entirely and promote ligand-independent growth [34]. In particular, AR-V7 expression in circulating tumor cells has been found in 19% and 39% of abiraterone and enzalutamide-treated patients, respectively [35]. AR-V7 expression in these patients has been associated with resistance to these agents [35].

While not as common as AR-V7 induction, point mutations that result in functional changes to the AR LBD are another mechanism of resistance to anti-androgen treatment strategies [36]. Examples of these mutations resulting in single amino acid changes are: H874Y, T877A, T877S, and W741L [36–38]. Recently, F877L mutations in the AR LBD have been described and are associated with enzalutamide resistance in both pre-clinical [10–12] models and clinical studies [11]. F877L mutations are also detected in a clinically-relevant number (5-10%) of enzalutamide-resistant patients [11, 13].

The F877L mutation results in a conformational change to the AR LBD [10] that abolishes the antagonist effect of enzalutamide. Further, under some circumstances, enzalutamide acts as an agonist on the mutant F877L AR [10]. Prior reports demonstrate that cessation of first-generation anti-androgens leads to anti-androgen withdrawal effects in as many as 25% of patients [9, 39–41]. However, anti-androgen withdrawal effects after discontinuing enzalutamide are only seen in a small percentage of patients, though the F877L mutation status of patients in that study was not reported [14]. Cohorts for whom AR mutational status is available in addition to PSA data after discontinuing enzalutamide will be necessary to understand clinically whether enzalutamide discontinuation can lead to an anti-androgen withdrawal effect like that seen with other anti-androgens. Nonetheless, a deeper understanding of determinants of the antagonist-to-agonist switch is critical for developing new treatments to target AR F877L mutations.

Our data shed new light on molecular mechanisms that contribute to mutant F877L AR activation by enzalutamide. While cells expressing the F877L mutation are resistant to enzalutamide, the enzalutamide agonistic effect on F877L AR is correlated with cellular androgen content. Indeed, we found that enzalutamide treatment of F877L-expressing MR49F cells only enhanced cell growth under low androgen conditions (charcoal-stripped serum). Furthermore, enzalutamide activates a transcriptional program that strongly overlaps with that activated by DHT, but induction of this program only occurred when androgens were depleted from culture. The effect of androgen interference with enzalutamide agonism was confirmed in an additional model – LNCaP cells with ectopic overexpression of F877L.

One explanation for the lack of enzalutamide agonism in androgen-replete conditions is that enzalutamide activation of mutant F877L AR is cancelled out by suppression of wild-type AR. Another possible explanation is that, in androgen-replete conditions, androgen ligands compete with enzalutamide for binding and activation of mutant F877L AR, thereby diminishing agonistic effects of enzalutamide. Indeed, a recent report demonstrates that intratumoral androgens persist or may increase in enzalutamide-resistant tumors [42]. Our data using both MR49F cells and PC3 cells grown in androgen-depleted conditions demonstrate that DHT concentrations similar to those achievable in human CRPC [21, 43] are better activators of mutant F877L AR than enzalutamide, further supporting our hypothesis that androgens compete for F877L AR activation.

In this study, we also measured the global gene expression change induced by DHT or enzalutamide treatment in F877L AR-expressing cells. DHT treatment induced a greater number of gene expression changes versus enzalutamide treatment, and the magnitude of gene expression change for nearly all genes was also greater with DHT vs. enzalutamide treatment. This further demonstrates that DHT may more potently activate F877L AR than enzalutamide.

Importantly, while DHT induced many unique gene expression changes vs. enzalutamide, nearly all the gene expression changes induced by enzalutamide were shared with DHT treatment. This demonstrates that enzalutamide agonism of F877L AR does not activate a distinct gene expression program vs. DHT. Further, there was a strong enrichment for a signature of canonical wild-type AR signaling [15] with either DHT or enzalutamide stimulation of MR49F cells, demonstrating that mutant F877L AR does not direct a distinct transcriptional program from wild-type AR.

Inhibition of BET bromodomain proteins was shown previously to block wild-type AR activation by androgens [15]. BET bromodomain proteins are chromatin readers that recognize acetyl lysine residues on histone tails and promote transcription of important genes in cancer, such as *c-Myc* and others, by cooperating with transcription factors, including: AR, the estrogen receptor, GATA1, and p53 [15, 22, 44–47]. Treatment with the BET bromodomain inhibitor JQ-1 interferes with ligand-induced activation of wild-type AR and blocks CRPC cell survival [15]. For this reason, we tested the BET bromodomain inhibitor JQ-1 in mutant F877L AR-expressing models. JQ-1 co-treatment blocked either DHT or enzalutamide-induced growth of MR49F cells and also blocked induction of AR target gene expression (Figure 3D, Supplementary Table S2). We also confirmed this effect in cells that only expressed mutant F877L AR, demonstrating that JQ-1 is an effective treatment to block F877L AR activation even in the absence of wild-type AR (Figure 3E).

Prior work demonstrated that AR-dependent cell lines are more susceptible to JQ-1 treatment than AR-null cell lines, suggesting that AR is a critical BET bromodomain inhibitor target in CRPC. [15]. We found that suppression of cell viability by JQ-1 was much greater under androgen-replete conditions compared to androgen-depleted conditions (Supplementary Figure S5). Thus, our results further confirm that suppression of ligand-dependent AR function is a key determinant of the anti-tumor activity of JQ-1 in CRPC models.

We also measured the effect of JQ-1 on growth of MR49F xenografts implanted in castrated mice *in vivo*. We determined that JQ-1 co-treatment with enzalutamide blocked tumor growth vs. enzalutamide treatment alone. Body weights were similar between the two treatment arms, and we did not observe any additional toxicity with JQ-1 + enzalutamide vs. enzalutamide alone. This further demonstrates the potential of BET bromodomain inhibition for the treatment of F877L AR-expressing CRPC tumors and the preliminary safety of combining BET bromodomain inhibitors with enzalutamide.

Altogether, our data demonstrate that the androgen content of the CRPC cell influences enzalutamide's ability to activate mutant F877L AR and suggest that enzalutamide agonism may be more pronounced in tumors harboring F877L mutations that have the lowest androgen concentrations. Finally, our results demonstrate that BET bromodomain inhibition is a promising treatment to block mutant F877L AR, irrespective of whether the AR ligand is androgens or enzalutamide. Clinical trials with BET bromodomain inhibition in CRPC that have recently begun (NCT02711956) will be necessary to confirm these results.

## MATERIALS AND METHODS

### Cell culture

PC3 cells were purchased from American Type Culture Collection (ATCC, Manassas, VA). LNCaP Pb. EGFP cells stably overexpressing AR^F877L^, AR^Wild-Type^, or empty control vector were a kind gift from Dr. Charles Sawyers [12]. All cells were maintained in RPMI1640 with 10% fetal bovine serum. Additionally, MR49F cells were maintained in media with 10 μM enzalutamide.

### Drug treatments

Enzalutamide was obtained from MedchemExpress (HY-70002). Dihydrotestosterone (DHT) was obtained from Sigma (A8380). For *in vitro* experiments, JQ-1 was obtained from BPS Bioscience (27402). For *in vivo* studies, JQ-1 was obtained from the laboratory of Dr. James Bradner, Dana Farber Cancer Institute Dept. of Medical Oncology/Harvard Medical School Dept. of Medicine. For *in vitro* studies, DMSO stocks of enzalutamide and JQ-1 and ethanol stocks of DHT were diluted to desired working concentrations in cell culture media (RPMI with 10% charcoal-stripped fetal bovine serum unless otherwise noted) with a final vehicle concentration of 0.1%. Vehicle-only controls were used in all cases, and vehicle concentrations were normalized across all drug co-treatments. See below for information on administration of drugs for *in vivo* studies.

### *In vivo* studies

MR49F xenografts were implanted in 7-8 week old castrated, immunocompromised male mice (Athymic Nude-*Foxn1^nu^*, Harlan Laboratories, strain code 069). Once tumors reached 100mm^3^, groups of mice were randomized to treatment with DMSO or JQ-1 (50 mg/kg) by intraperitoneal injection Monday through Friday. All animals were treated with enzalutamide (10 mg/kg) by oral gavage Monday through Friday. Tumor measurements and body weight measurements were recorded, and all animals were sacrificed on Day 28. Tumor volumes and final body weights are shown as mean ± standard error of the mean (SEM). 2-sided t-test with equal variance was performed using end point (week 4) data. Differential tumor wet weights at sacrifice between treatment groups was tested using a 2-sided 2-sample t-test with Welch's correction, as the distribution appeared to be approximately normal. P-values less than 0.05 were considered statistically significant. Mice were housed under specific pathogen-free conditions (5 mice per shoebox cage) and maintained by the OHSU Department of Comparative Medicine. All *in vivo* studies were conducted under an OHSU IACUC-approved protocol (protocol #IS00003757).

### Immunoblotting

Experiments were performed as described previously [48], using primary antibodies to AR (Santa Cruz sc-816X and Millipore 06-680), PSA (Abcam ab53774), and β-Actin (Sigma A5441). Blots were imaged using the LI-COR Odyssey imaging system according to the manufacturer's instructions.

### RNA Isolation and RT-qPCR

Cells were lysed using Trizol reagent (Life Technologies), and RNA was isolated using chloroform extraction and alcohol precipitation per manufacturer's instructions. RNA concentration was determined using a NanoDrop ND-1000 spectrophotometer. 1.0 μg RNA was reverse-transcribed using a High Capacity cDNA Reverse Transcription Kit (Life Technologies) with random primers. RT-qPCR was performed using a 7500 Fast thermocycler (Life Technologies) with the following cycling program: 50°C for 2 min, 95°C for 10 min, 40 cycles of 95°C for 15 sec dissociation, 60°C for 1 min annealing/extension/read. 10 μL singleplex RT-qPCR reactions contained 1X TaqMan (Life Technologies) universal standard Master Mix, 1X TaqMan hydrolysis probe, and 10 ng RNA-equivalent cDNA template. TaqMan probes were used to detect human *KLK3* (Hs02576345_m1), *TMPRSS2* (Hs01120965_m1), *NKX3.1* (Hs00171834_m1). Human *β-actin* TaqMan probe (Hs99999903_m1) was used as an endogenous control. RT-qPCR raw Ct data were analyzed with 7500 Software v2.0.5 and DataAssist Software v3.0 (Life Technologies).

### RNA-seq library preparation and expression analysis

Total RNA was extracted with Trizol/CHCl_3_ (Life Technologies) according to the manufacturer's protocol. Two wells were combined for each replicate for RNA extraction in 1 mL Trizol, and the aqueous phase was put through the Qiagen RNEasy kit for cleanup. Sample preparation followed the Agilent SureSelect Strand-Specific mRNA Library Preparation Protocol (Version A.2, September 2013, Agilent Technologies). Poly-A RNA was purified from 1 μg total RNA per sample using two serial rounds of binding to oligo dT magnetic beads. The poly(A) RNA was chemically-fragmented, and first-stand cDNA was synthesized using the RNA-seq First Strand Master Mix (Agilent). After purifying the first strand cDNA using AMPure XP beads, second-strand cDNA was synthesized and ends were repaired. A second round of cDNA purification with AMPure XP beads occurred, and the 3′ ends were adenylated, followed by adapter ligation and AMPure XP beads purification. Ligated DNA was PCR-amplified for 14 cycles and purified again with AMPure XP beads. Quality of the resulting libraries was assessed with an Agilent 2100 Bioanalyzer DNA 1000 Assay. Libraries were sequenced on an Illumina HiSeq as single-end 50 bp reads in the OHSU Massively Parallel Sequencing Shared Resource.

RNA-seq was conducted on three biological replicates of each cell line/drug treatment. RNA-seq data analysis was performed using the Tuxedo Suite [49–51]. Each sample was mapped independently to the human genome build GRCh37/hg19 using Tophat v2.0.9 [49, 50]. Transcript assembly and quantification was done with Cufflinks v2.1.1 [50, 51] or HTSeq v0.6.1 [52]. For genes with multiple transcripts, the estimates of transcript expression were summed to yield a single estimate of gene-level expression in FPKM units. Sequence data are available at Gene Expression Omnibus with accession number GSE69896.

To identify genes that were differentially-expressed compared to mock treatment, data was first filtered to remove genes with low variation across the sample set. For each cell line, we used a t-test on the filtered normalized expression values to identify genes whose expression was significantly changed by drug treatment (enzalutamide, DHT, and/or JQ-1). We used Benjamini-Hochberg false discovery rate to account for multiple comparisons, and deemed genes with q < 0.05 to be significant. Statistical analyses were performed in R (version 3.1.2) and displayed in RStudio (version 0.98.501).

Gene Set Enrichment Analysis (GSEA) [53] was run using RNA-seq gene expression data from triplicate cell line samples treated with vehicle or drug. Specifically, the expression data were interrogated for enrichment in the gene set of interest: an AR expression signature described previously [15]. The expression data were permutated 1000 times to generate the resulting GSEA plots, normalized enrichment scores, and FDR q-values (Supplementary Figure S2).

Gene ontology (GO) enrichment and functional annotation was performed on the 358 genes concordantly differentially expressed with enzalutamide and DHT treatment from Figure 2E, and the 57 shared enzalutamide and DHT-regulated genes from Figure 3A whose expression is significantly reversed (≥2 fold) by JQ-1 using the Database for Annotation, Visualization and Integrated Discovery (DAVID, v6.7). Specifically, the genes of interest were examined against Gene Ontology Biological Process, Molecular Function, and Cellular Component curated gene sets (levels 4 and 5), and Biocarta, Kegg, Reactome, Panther and the Biological Biochemical Image pathway databases.

### Plasmids

The ARE-4 firefly luciferase reporter of AR transcriptional activation has been reported previously and was a kind gift from Dr. Xin Yuan [23, 24]. For normalization of firefly luciferase values, cells were co-transfected at a 1:10 ratio with a plasmid constitutively expressing *Renilla* luciferase, driven by the SV40 promoter. This plasmid was a kind gift from the laboratory of Dr. David Qian of the OHSU Knight Cancer Institute.

### Transfection of siRNA

To transiently knock down AR, cells were transfected with AR siRNA oligonucleotides 5′-(GACCUACCGAGGAGCUUUCdTdT-3′) (Dharmacon) described previously [54]. Control siRNA oligonucleotides targeting luciferase (siNTC: 5′-CGUACGCGGAAUACUUCGAdTdT-3′) were transfected in parallel. Cells were transfected with Lipofectamine 2000 (Life Technologies) per manufacturer's recommendation to a final concentration of 50 nM siRNA.

### Cell viability assays

MTS assays of viability were performed using the CellTiter 96^®^ Aqueous One Solution Cell Proliferation Assay System (Promega) per the manufacturer's instructions. Counts of viable cells were performed using the trypan blue exclusion method as calculated by the Countess instrument (Life Technologies) according to the manufacturer's instructions.

### Flow cytometry

LNCaP Pb. EGFP AR^Wild-Type^ or AR^F877L^ overexpressing cells were cultured in either full-serum or charcoal-stripped medium as described above for one day prior to drug treatment. Drug treatments were then performed in the appropriate serum (either full- or charcoal-stripped) as described above for three days. Cells were dissociated with Accumax reagent (Stem Cell Technologies). GFP reporter activity in live cells was measured with flow cytometry Core using a BD FACS Canto II instrument (BD Biosciences) in the OHSU Flow Cytometry Core. The resultant FCS files were analyzed and plotted using FlowJo Single Cell Analysis Software.

### Luciferase assays

PC3 cells were transiently co-transfected with equal amounts of an AR F877L mutant expression construct and a 10:1 ratio of ARE4-Luc AR firefly luciferase reporter [23, 24] and constitutive pSV40-Renilla luciferase expression plasmid as has been described previously. A total of 2 μg DNA per well of a 6-well plate was transfected using Lipofectamine 2000 according to manufacturer's protocol. Luminescence measurements were obtained using a Promega Dual Luciferase Assay System and Veritas microplate illuminometer (Turner Biosystems) according to manufacturers' instructions. Firefly luciferase reporter activity was normalized to Renilla values for all samples.

### Statistics

Statistical analyses of RNA-seq data are described in detail above, and the number of replicates is described in the above methods or the respective figure legends. Statistical analyses of *in vivo* experiments is also described in detail above, and values of error bars and number of replicates is indicated in the Figure 4 legend. Statistical comparisons of all other experiments were performed using a Student's two-tailed t-test with equal variance. Values of error bars and number of replicates are indicated in the respective figure legends.

## SUPPLEMENTARY FIGURES AND TABLES












