# Supplementary material for: Cellular androgen content influences enzalutamide agonism of F877L mutant androgen receptor
Source: Oncotarget. 2016 Jun 3;7(26):40690–703. doi: 10.18632/oncotarget.9816 (PMC5130036; doi:10.18632/oncotarget.9816)
Supplement: Supplementary file 1 [file oncotarget-07-40690-s001.pdf]

Cellular androgen content influences enzalutamide agonism of F877L mutant androgen receptor

SUPPLEMENTARY FIGURES AND TABLES

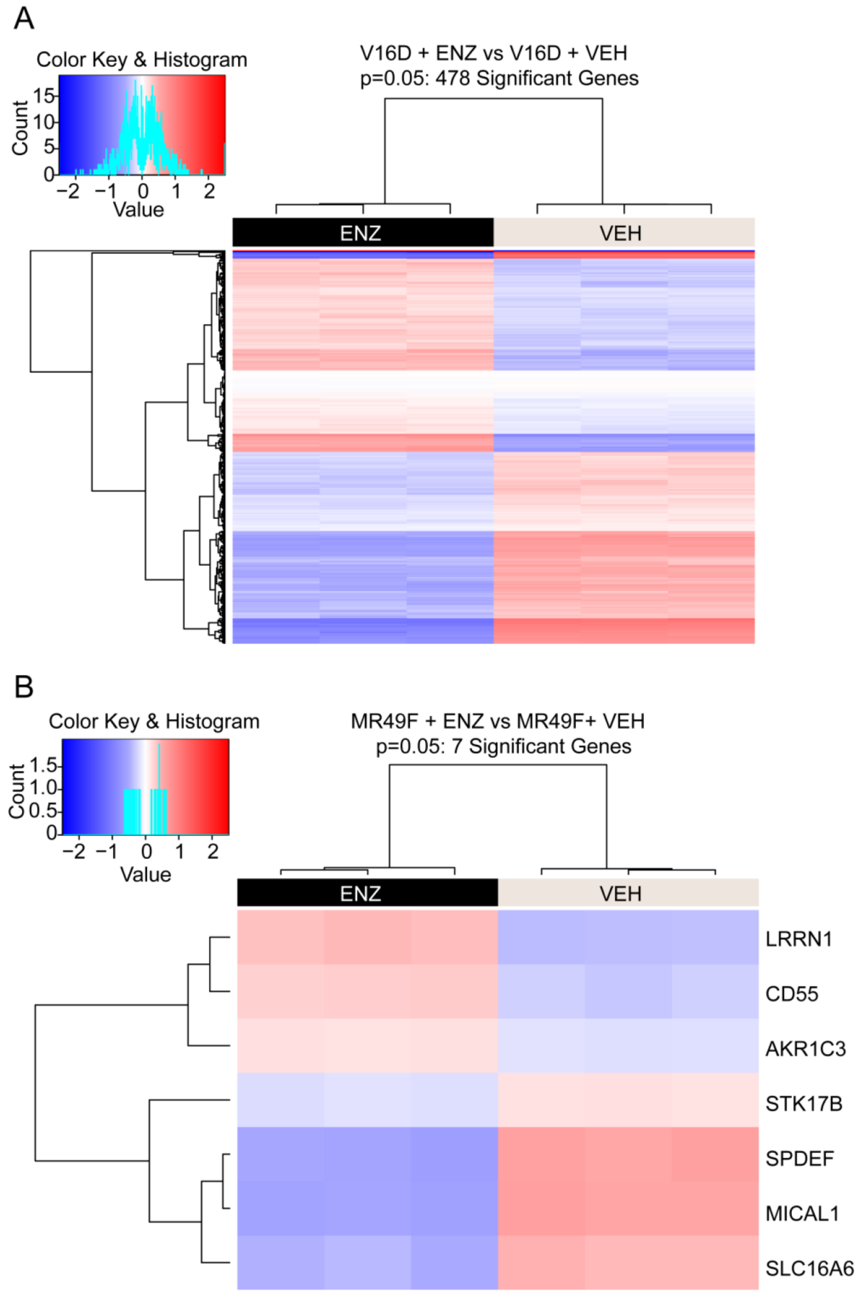

Supplementary Figure S1: Gene expression changes after enzalutamide treatment of V16D or MR49F cells grown in androgen-replete conditions. Heat map of RNA-seq data (see Figure 1E). **A.** There are 478 significant gene expression changes after 24 hour enzalutamide (10 µM) treatment of V16D cells. **B.** In contrast, only seven genes showed significant expression changes after enzalutamide treatment of MR49F cells. Expression data per gene represents the mean log2-transformed FPKM values of three biological replicates. After filtering based on variance, we used a t-test to determine significant differentially expressed genes in the enzalutamide-treated vs. vehicle-treated condition (FDR-adjusted p-value ≤ 0.05).

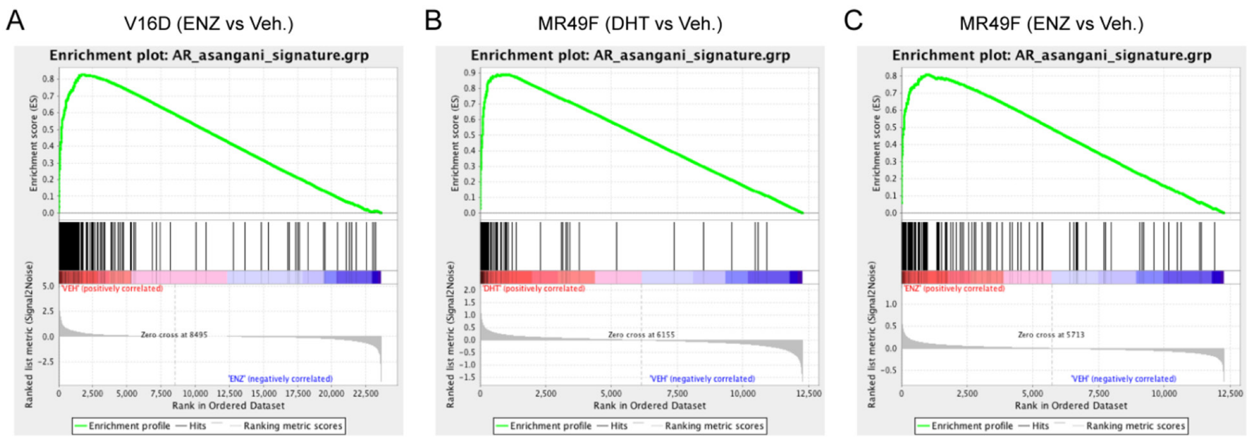

Supplementary Figure S2: Gene set enrichment analysis demonstrates strong enrichment of an AR transcriptional signature with enzalutamide or DHT treatment. Gene set enrichment analysis (GSEA) was performed on RNA-seq data. **A.** V16D cells treated with 10 µM enzalutamide for 24 hours (see Figure 1E, Figure S1A) showed a significant enrichment (normalized enrichment score = -1.24, q-value = 0.096) for a previously reported AR target gene signature (15) in comparison to vehicle treatment. **B, C.** MR49F were cells grown in androgen-depleted medium and treated with either (B) 1 nM DHT or (C) 10 µM enzalutamide for 24 hours (see Figure 2E). For both treatments, there was significant enrichment (NES = 1.02, 1.088 and q-values=0.099, 0.092 respectively) with the previously reported AR target gene signature (15). GSEA analysis was run on genome-wide expression data representing 3 biological replicates per treatment condition with 1000 phenotype permutations against the AR target gene signature of interest (15).

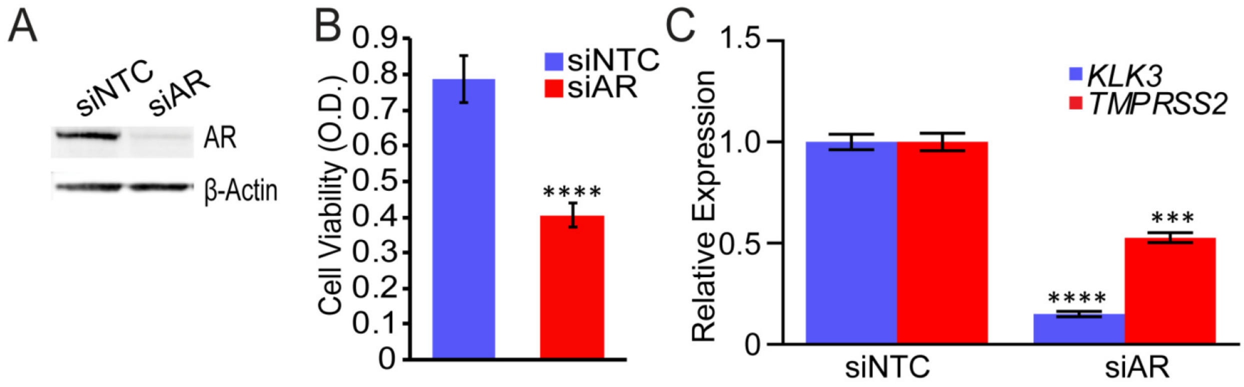

Supplementary Figure S3: Enzalutamide-resistant MR49F cells are still AR-dependent. MR49F cells were grown in full serum supplemented with 10 µM enzalutamide. **A.** siRNA was used to knockdown AR, and samples were collected 96 hours later. **B.** Cell viability was measured using MTS assays, and **C.** expression of AR targets was measured by RT-qPCR. MTS data are the means of 12 biological replicates; error bars represent standard deviations. **** = p≤.0001, unpaired 2-tailed t-test. RT-qPCR data are mean RQ (ΔΔCt method) of three biological replicates; positive and negative error bars represent SEM. *** = p≤0.001, **** = p≤0.0001, unpaired 2-tailed t-test. Comparisons are between siNTC and siAR.

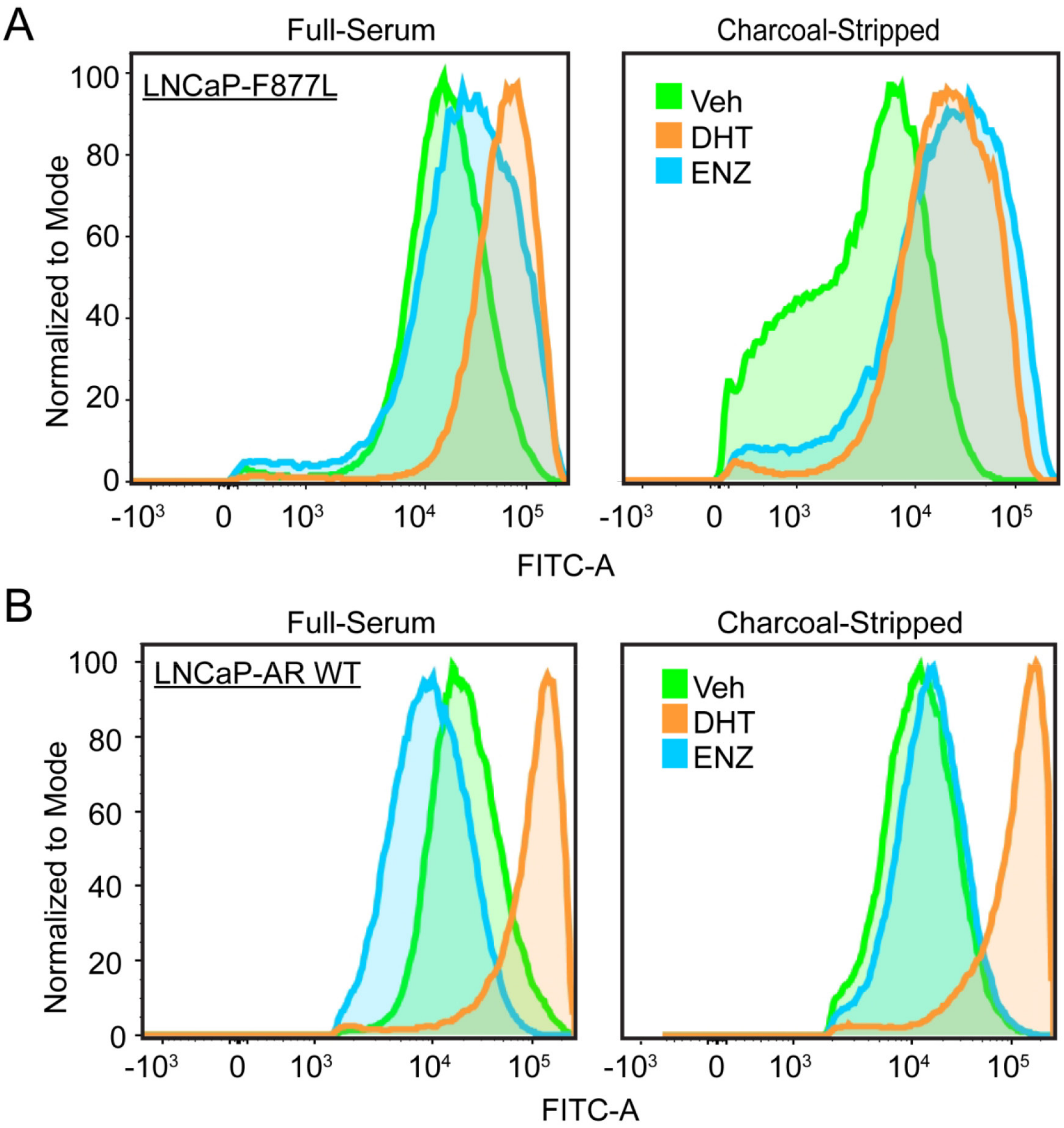

Supplementary Figure S4: Enzalutamide agonism of AR reporter activity is restricted to mutant F877L AR. A. Flow cytometry histograms of LNCaP-F877L cells from Figure 2E grown in either androgen-replete serum or androgen-depleted, charcoal-stripped serum. **B.** Flow cytometry histograms of LNCaP cells stably expressing ectopic wild-type AR and the probasin-GFP reporter.

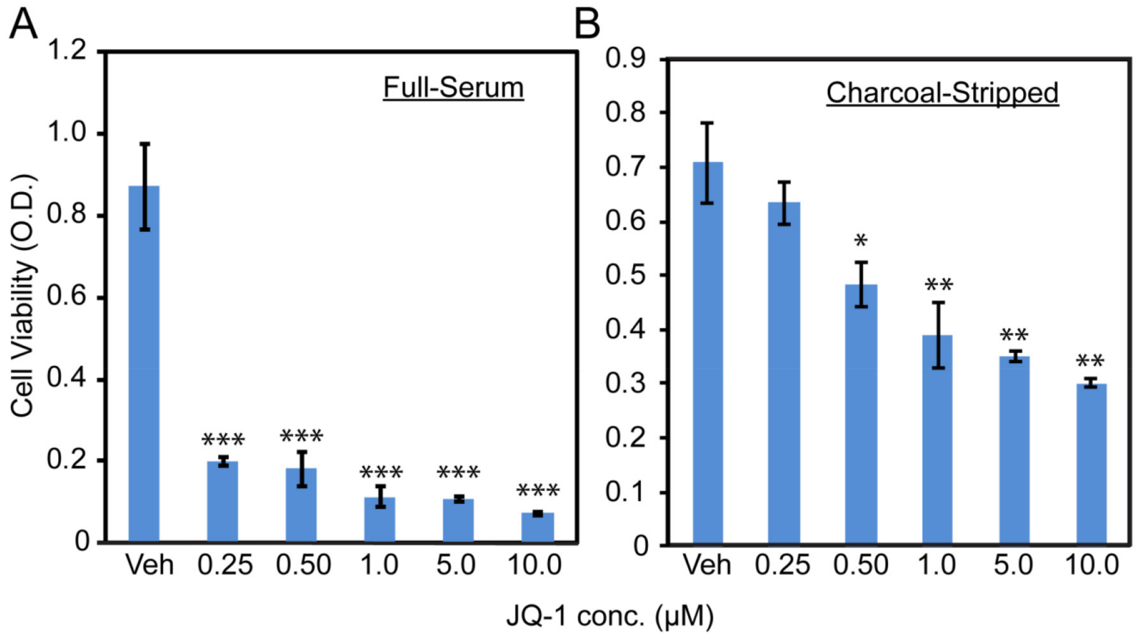

Supplementary Figure S5: The growth-inhibitory effects of JQ-1 treatment are most pronounced in androgen-replete conditions. MTS assay of viability in MR49F cells grown in androgen-replete serum **A.** or charcoal-stripped serum **B.** treated with dose escalation of JQ-1 for 6 days. Data are mean of three biological replicates; positive and negative error bars represent standard deviations. * = p≤0.05, ** = p≤0.01, *** = p≤0.001, unpaired 2-tailed t-test.

Supplementary Table S1: Top 10 gene ontology (GO) categories enriched in the 358 conserved, differentially-expressed genes after DHT or enzalutamide treatment

Category	Term	p-value
SP_PIR_KEYWORDS	DNA replication	1.10E-14
GOTERM_BP_5	GO:0006260~DNA replication	4.40E-14
GOTERM_BP_5	GO:0006259~DNA metabolic process	7.54E-13
GOTERM_BP_4	GO:0006259~DNA metabolic process	7.55E-13
REACTOME_PATHWAY	REACT_383:DNA replication	1.88E-11
SP_PIR_KEYWORDS	Cell cycle	3.18E-11
REACTOME_PATHWAY	REACT_152:Cell Cycle, Mitotic	1.77E-10
UP_SEQ_FEATURE	Domain:MCM	7.54E-10
INTERPRO	IPR018525:DNA-dependent ATPase MCM, Conserved site	1.01E-09
SP_PIR_KEYWORDS	ATP-binding	1.51E-09

Supplementary Table S2: Top 10 gene ontology (GO) categories enriched in the 57 genes with at least a 2-fold change in expression with JQ-1 co-treatment vs. treatment with either enzalutamide or DHT alone

Category	Term	p-value
GOTERM_BP_5	GO:0006260~DNA replication	2.69E-05
SP_PIR_KEYWORDS	DNA replication	1.17E-04
GOTERM_BP_5	GO:0006259~DNA metabolic process	1.55E-04
GOTERM_BP_4	GO:0006259~DNA metabolic process	1.98E-04
UP_SEQ_FEATURE	Sequence variant	0.004585
GOTERM_BP_5	GO:0051052~Regulation of DNA metabolic process	0.005569
GOTERM_BP_4	GO:0051052~Regulation of DNA metabolic process	0.006056
SP_PIR_KEYWORDS	Polymorphism	0.008224
SP_PIR_KEYWORDS	Cell cycle	0.010069
REACTOME_PATHWAY	REACT_383:DNA Replication	0.011466
